# CRISPR/Cas9 Editing of the Polyomavirus Tumor Antigens Inhibits Merkel Cell Carcinoma Growth In Vitro

**DOI:** 10.3390/cancers11091260

**Published:** 2019-08-28

**Authors:** Arturo Temblador, Dimitrios Topalis, Graciela Andrei, Robert Snoeck

**Affiliations:** Laboratory of Virology and Chemotherapy, Department of Microbiology, Immunology and Transplantation, Rega Institute for Medical Research, KU Leuven, 3000 Leuven, Belgium

**Keywords:** Merkel cell carcinoma, Merkel cell polyomavirus, viral tumor antigens, CRISPR/Cas9, cell cycle regulation, novel therapeutics

## Abstract

Merkel cell carcinoma (MCC) is an aggressive type of skin cancer whose main causative agent is Merkel cell polyomavirus (MCPyV). MCPyV is integrated into the genome of the tumor cells in most MCCs. Virus-positive tumor cells constitutively express two viral oncoproteins that promote cell growth: the small (sT) and the large (LT) tumor antigens (TAs). Despite the success of immunotherapies in patients with MCC, not all individuals respond to these treatments. Therefore, new therapeutic options continue to be investigated. Herein, we used CRISPR/Cas9 to target the viral oncogenes in two virus-positive MCC cell lines: MS-1 and WAGA. Frameshift mutations introduced in the target sequence upon repair of the Cas9-induced DNA break resulted in decreased LT protein levels, which subsequently impaired cell proliferation, caused cell cycle arrest, and led to increased apoptosis. Importantly, a virus-negative non-MCC cell line (HEK293T) remained unaffected, as well as those cells expressing a non-targeting single-guide RNA (sgRNA). Thus, we presumed that the noted effects were not due to the off-target activity of the TAs-targeting sgRNAs. Additionally, WAGA cells had altered levels of cellular proteins involved in cell cycle regulation, supporting the observed cell cycle. Taken together, our findings provide evidence for the development of a CRISPR/Cas9-based therapeutic option for virus-positive MCC.

## 1. Introduction

Merkel cell carcinoma (MCC) is a rare but aggressive type of skin cancer with increasing incidence, currently at 0.7 cases per 100,000 individuals in the US [1]. Major factors associated with MCC are UV-light exposure in fair-skinned people, immunosuppression, and presentation in the elderly [2]. Over the past few years, there has been a growing interest in MCC due to the identification of a new human polyomavirus, Merkel cell polyomavirus (MCPyV), clonally integrated into ~80% of MCCs [3]. Nevertheless, MCPyV-negative (MCPyV^−^) MCCs are a smaller fraction of tumors characterized by a higher mutational burden with UV-light signature [4].

Polyomaviruses are non-enveloped viruses with a dsDNA genome of ~5 kb divided into an early and late region separated by a non-coding control region (NCCR). MCPyV-positive (MCPyV^+^) MCCs constitutively express two alternatively spliced products of the early gene: the small (sT) and the large (LT) tumor antigens (TAs) [5]. During the normal viral life cycle, the TAs stimulate S phase entry, so that the virus can hijack the host cell replication machinery to replicate its own genome [6]. Though MCPyV infection is widespread [7], MCC is a dead-end for MCPyV replication, as truncating mutations in the LT disrupt the origin binding and helicase/ATPase domains [8]. Nevertheless, the retinoblastoma (Rb) binding domain, necessary to promote cell growth and tumor progression, is conserved [9]. Among the diverse functions of MCPyV sT [10,11,12,13,14,15,16,17], the LT-stabilization domain (LSD) avoids degradation of the LT and cellular oncoproteins [18].

The five-year relative survival of patients diagnosed with metastatic MCC is as low as 18% [19]. Recently, the FDA has granted approval to avelumab and pembrolizumab as preferable treatments for patients with metastatic MCC, owing to their advantages over the classic chemotherapies. However, not all patients respond to these treatments and some develop resistance. Even when diagnosed at early stages, patients may be ineligible for surgery or radiotherapy due to other comorbidities. Consequently, new therapeutic options are needed [20].

Using short hairpin RNAs (shRNAs), Houben et al. showed that MCPyV TAs are required for the maintenance of MCPyV^+^ MCC cells [21,22]. Since MCPyV is integrated into the genome of the tumor cells, the use of gene-editing tools is a promising therapeutic strategy for MCC. In recent years, the CRISPR/Cas9 system has revolutionized the genome-engineering field [23]. It consists of the *Streptococcus pyogenes* Cas9 (*Sp*Cas9) endonuclease and a single-guide RNA (sgRNA) that contains a spacer sequence matching a target site next to the protospacer adjacent motif (PAM) [24]. In the absence of a template, the cellular DNA repair machinery resolves the Cas9-induced DNA break by non-homologous end joining (NHEJ), an error-prone mechanism that generates insertions and deletions (indels) in the target sequence [25]. Contrary to shRNAs that reduce gene expression transitorily, CRISPR/Cas9 editing could generate stable and permanent changes in the genomic sequence of MCPyV TAs. This approach has been successfully used to eliminate JCPyV infection [26].

In the present study, we used CRISPR/Cas9 to introduce frameshift mutations in the genomic sequence of MCPyV TAs. Inactivation of the TAs affected cell proliferation, led to cell cycle arrest, increased apoptosis and changed the expression of cellular proteins involved in cell cycle regulation. Importantly, a MCPyV^−^-non-MCC cell line remained unaffected, as well as those cells expressing a non-targeting sgRNA.

## 2. Results

### 2.1. CRISPR/Cas9 Editing Induced Mutations in MCPyV TAs Genomic Sequence

**Figure 1:**
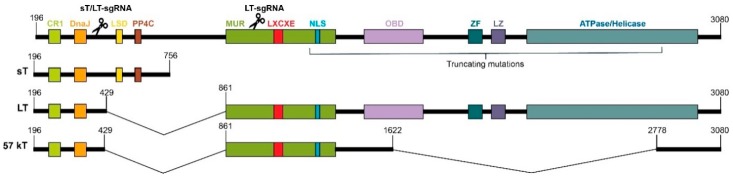
Schematic representation of functional domains of Merkel cell polyomavirus (MCPyV) tumor antigens (TAs). The small, large and 57 kDa tumor antigens (sT, LT, and 57 kT, respectively) are alternatively spliced products of the early gene of MCPyV. MCPyV TAs share their N-terminal region (exon 1), comprising the conserved region 1 (CR1) and DnaJ domains. Unique regions comprise: LSD (LT stabilization domain) and PP4C (protein phosphatase 4 catalytic subunit) for sT and MUR (MCPyV unique region), LXCXE (Rb-binding domain), NLS (nuclear localization signal), OBD (origin-binding domain), ZF (zinc finger), LZ (leucine zipper), and ATPase/Helicase domain for LT. The region that harbors the truncating mutations is demarcated and the target site of each sgRNA is indicated with scissors. Numbers specify nucleotide position in the MCPyV genome. Adapted from Harms et al., 2018 [20].

**Figure 2:**
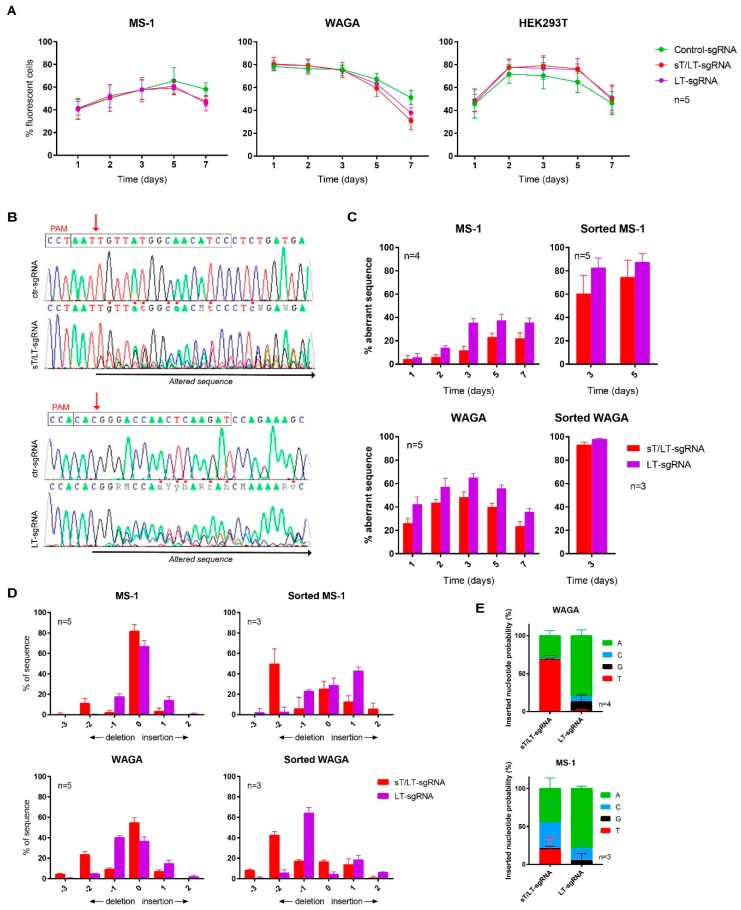
CRISPR/Cas9 editing induced mutations in MCPyV TAs genomic sequence. (**A**) Evolution of OFP fluorescence during the seven days post-transfection; (**B**) Representative electropherograms of MCPyV TAs genomic sequences after CRISPR/Cas9 editing with the indicated sgRNAs. Boxes delimit the PAM sequence and the sgRNAs target sites. The red arrows denote the Cas9 cleavage position while the black left-right arrow indicates the mutated sequence; (**C**) Results of Tracking of Indels by DEcomposition (TIDE) analysis in sequences from cells expressing Cas9 and the specified sgRNAs. Sequences from cells expressing the ctr-sgRNA were used as reference control; (**D**) Indels distribution in genomic sequences from CRISPR/Cas9-targeted cells; (**E**) Probabilities (%) of insertion of each nucleotide in cells expressing each one of the sgRNAs. Data represent mean values ± SD and the number of independent experiments is indicated (n).

CRISPR/Cas9 editing of MCPyV TAs was assessed in two MCPyV^+^ MCC cell lines: MS-1 and WAGA. The GeneArt™ vector was used to obtain three constructs encoding Cas9 and a specific sgRNA (sT/LT-sgRNA, LT-sgRNA, or a non-targeting control, ctr-sgRNA). Figure 1 shows the sgRNAs target regions and functional domains of MCPyV TAs. An orange fluorescent protein (OFP) marker enabled the assessment of transfection efficiency over time (Figure 2A). Owing to their high transfectability, HEK293T cells served as the control for off-target activity of the targeting sgRNAs.

Cas9-induced breaks promoted the emergence of mutations in MCPyV^+^ cells expressing the TAs-targeting sgRNAs, but not with the ctr-sgRNA (Figure 2B). To estimate the percentage of aberrant DNA, sequencing results were analyzed by TIDE (Tracking of Indels by DEcomposition) [27]. The percentage of mutations progressively increased in both cell lines, MS-1 and WAGA, until it started to decline (Figure 2C), presumably owing to the loss of the vector as evidenced by decreased OFP fluorescence (Figure 2A). In OFP^+^-sorted cells, nearly 100% of DNA from WAGA cells was altered by CRISPR/Cas9. Sorted MS-1 cells also showed a considerably high percentage of mutations. Figure 2D illustrates the distribution of those mutations: the sT/LT-sgRNA mostly generated a two-base deletion while the LT-sgRNA was repaired mainly with a single-base deletion/insertion. The probabilities of insertion of each nucleotide are shown in Figure 2E: while they were more variable for the sT/LT-sgRNA, the LT-sgRNA induced the insertion of an adenine in ~80% of the cases.

### 2.2. Immunoblotting Confirmed Downregulation of MCPyV LT Protein

**Figure 3:**
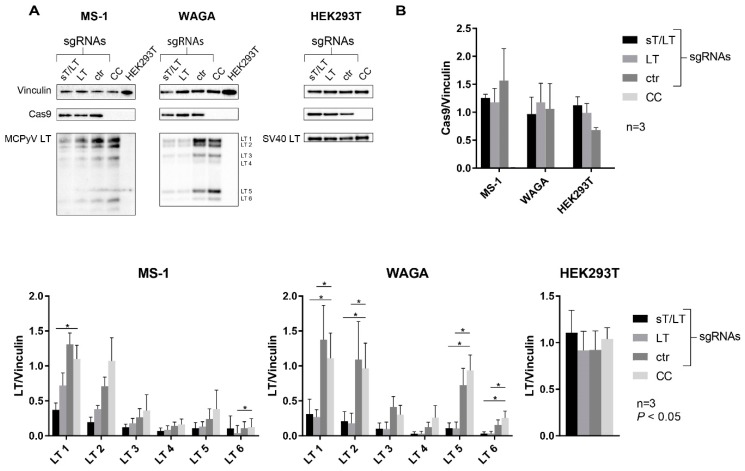
CRISPR/Cas9-mediated downregulation of MCPyV LT in MCPyV-positive (MCPyV^+^) MCC cells expressing TAs-targeting sgRNAs. (**A**) LT expression was analyzed by western blot using total protein extracts from MCPyV^+^ (MS-1 and WAGA) and MCPyV^−^ (HEK293T) cells expressing Cas9 endonuclease (detected with an anti-V5 antibody) and the indicated sgRNA. Non-transfected cells (cell control or CC and HEK293T) were used as controls. Representative blots of three independent experiments are shown. Vinculin was used as internal loading control; (**B**) Densitometry analysis of normalized Cas9/Vinculin and LT/Vinculin. As indicated in panel A, for MCPyV^+^ cells the different LT isoforms are enumerated from 1 to 6. Data represent mean values ± SD fromthree independent blots. Statistical significance compared with cell control (CC) is indicated.

MCPyV^+^ MCC cell lines harbor truncating mutations that lead to premature stop codons. Thus, WAGA cells express a truncated LT form with a predicted molecular mass of 30 KDa, while MS-1 truncated LT has 47 KDa. However, immunoblotting with a CM2B4 antibody shows multiple bands arising from post-translational modifications and aberrant splicing events [21,28]. As shown in Figure 3A, WAGA cells had a strong downregulation of all LT isoforms when expressing the TAs-targeting sgRNAs at day 3 post-transfection. For MS-1, the downregulation was more evident at day 5, but less pronounced than in WAGA. None of the MCPyV^+^ cell lines expressing the ctr-sgRNA changed LT protein levels when compared with a non-transfected cell control. HEK293T cells express the SV40 LT, which was not affected by the TAs-targeting sgRNAs. Figure 3B shows the densitometry analysis of each blot.

Since there is no commercially available antibody to detect MCPyV sT, a RT-qPCR assay was performed in WAGA cells. The sample expressing the ctr-sgRNA served as the calibrator: its 2^−ΔΔCt^ value was set to 1 and the fold-change expression was calculated for the other samples. RT-qPCR results showed a tendency for the reduced expression of the TAs when the sT/LT-sgRNA was used and only reduced expression of LT with the LT-sgRNA. Nevertheless, the results of four independent experiments did not give a significant difference, except for LT when targeted with the LT-sgRNA (Figure S1).

### 2.3. CRISPR/Cas9 Editing of MCPyV TAs Impaired Cell Proliferation

**Figure 4:**
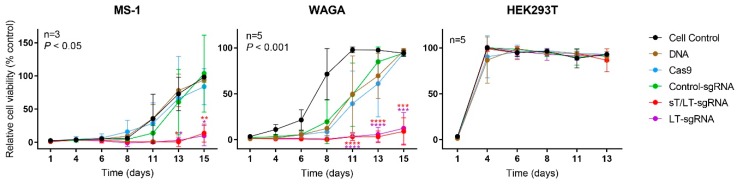
CRISPR/Cas9 editing of MCPyV TAs impaired cell proliferation. The day after transfection, cells were seeded in 96-well plates and growth was monitored over time using the WST-1 assay. Different control conditions were used: non-transfected cells (CC, cell control), cells transfected with an empty pUC19 plasmid (DNA), cells transfected with a plasmid expressing only Cas9 (Cas9), cells transfected with the CRISPR/Cas9 vector expressing a non-targeting sgRNA (ctr-sgRNA) and HEK293T cells transfected with all the targeting and control constructs. Cell proliferation is illustrated as a percentage relative to the cell control at 100% viability. Data are represented as mean values ± SD. The number of independent experiments is indicated (n) as well as statistical significance (*P*-values) when compared with the cell control (CC).

Proliferating cells were quantified using the WST-1 colorimetric assay. Two additional controls were used: cells transfected with an empty pUC19 vector (DNA) or cells expressing only Cas9 (Cas9). As depicted in Figure 4, the proliferation of cells expressing the TAs-targeting sgRNAs was impaired in MCPyV^+^ cells, when compared with the controls. Conversely, this effect was not observable in HEK293T cells, which are MCPyV^−^. Cells transfected with the control constructs had a considerable steady phase following transfection in comparison to the exponential proliferation of cell control.

### 2.4. CRISPR/Cas9 Editing of MCPyV TAs Resulted in Cell Cycle Arrest and Cell Death

**Figure 5:**
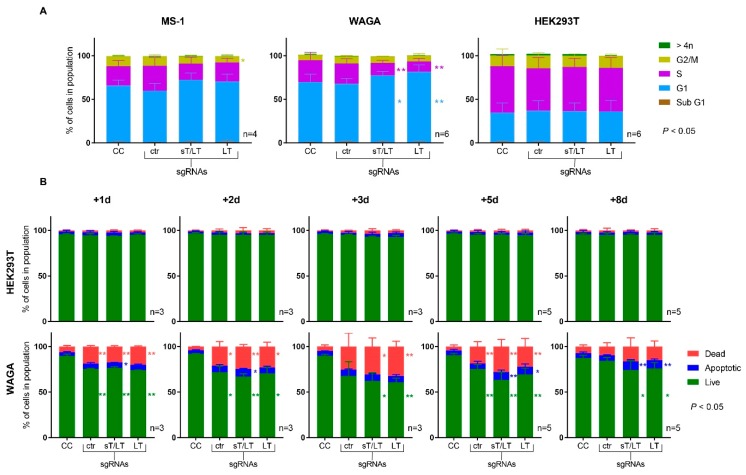
Cell cycle distribution and apoptosis analysis upon CRISPR/Cas9 editing of MCPyV TAs. (**A**) Five days after transfection with the indicated CRISPR/Cas9 constructs (ctr-sgRNA, sT/LT-sgRNA, or LT-sgRNA), cell nuclei were stained with PI to analyze cell cycle distribution. The plots depict the percentage of cells in each phase of the cell cycle; (**B**) Cells were stained for apoptosis analysis (Annexin V-APC/viability dye). According to flow cytometry results, cell fractions were separated in live (Annexin V-APC^−^/viability dye^−^), dead (Annexin V-APC^+^/viability dye^+^), or apoptotic (Annexin V-APC^+^/viability dye^−^). Data are represented as mean values ± SD. The number of independent experiments is indicated (n) as well as the statistical significance (*p* < 0.05) when compared with the cell control (CC).

The cellular DNA content of transfected cells was analyzed by propidium iodide (PI) staining of cell nuclei at day 5 post-transfection (Figure 5A). Expression of the TAs-targeting sgRNAs in WAGA cells led to a reduction in cell cycle progression, as evidenced by the significant larger fraction of cells in G1 and the decreased fraction in the S phase. However, a significant difference was only observed in the G2/M phase of MS-1 cells expressing the LT-sgRNA. No alterations in the cell cycle were noted in cells expressing the ctr-sgRNA or in HEK293T cells.

Figure 5B depicts the cell death profiles in WAGA and HEK293T cells at 1, 2, 3, 5, and 8 days post-transfection. Shortly after transfection, WAGA cells expressing the ctr-sgRNA suffered cell death. Nevertheless, they progressively recovered to reach the level of the cell control. Conversely, expression of the TAs-targeting sgRNAs resulted in a significant increase of apoptotic cells (Annexin V-APC^+^/viability dye negative), when compared with the cell control, at day 8 after transfection. The TAs-targeting sgRNAs induced cell death to a similar degree. Moreover, they caused a significant reduction in the number of viable cells and an increase in necrotic cells. These differences were not observable in HEK293T cells, which showed an invariable prominent fraction of living cells. As shown in Figure S2, the activation of caspase 3 following CRISPR/Cas9 targeting was investigated by western blot and compared with the cell control. The results showed that this cell death pathway is not activated by loss of MCPyV TAs.

### 2.5. Altered Expression of Cell Cycle Regulatory Proteins upon CRISPR/Cas9 Editing of MCPyV TAs

**Figure 6:**
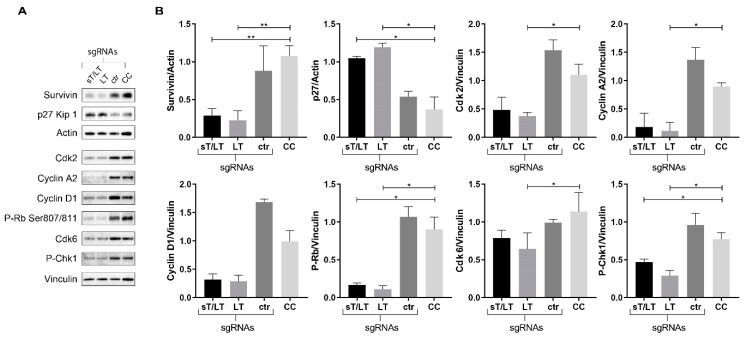
Altered cell cycle regulation upon CRISPR/Cas9 editing of MCPyV TAs. (**A**) Blots, representative of three independent experiments, of total protein extracts from WAGA cells expressing the indicated constructs and non-transfected cells (CC) as controls. Actin and Vinculin were used as internal loading controls; (**B**) Densitometry analysis of blots normalized with actin or vinculin. Data represent mean values ± SD fromthree independent blots. Statistical significance compared with cell control (CC) is indicated.

To explore which proteins involved in cell cycle regulation contributed to the cell cycle arrest observed in MCPyV^+^ cells, immunoblot analysis of key regulators was performed. Total protein extracts of WAGA cells were used, given the efficient CRISPR/Cas9 editing of the TAs. MCPyV LT has been shown to regulate the transcriptional activation of survivin, an anti-apoptotic protein [29]. Immunoblot analysis of survivin revealed a significant decrease upon TAs targeting (Figure 6A,B). TAs targeting resulted in a significant increase in the negative regulator p27. Conversely, levels of proteins mostly involved in G1 to S phase transition (Cdk2, Cdk6, cyclin A2, cyclin D2, and P-Chk1), were downregulated. Moreover, Rb phosphorylation at serines 807/811 was significantly decreased upon TAs targeting (Figure 6A). For Cdk2, Cyclin A2 and Cdk6, the results were only significant when using the LT-sgRNA. Importantly, no alterations were observed in extracts from cells expressing the ctr-sgRNA.

## 3. Discussion

In the present study, we investigated the potential inactivation of MCPyV TAs using CRISPR/Cas9 editing in two MCPyV^+^ MCC cell lines, MS-1, and WAGA. The efficacy of CRISPR/Cas9 to induce mutations varied according to the sgRNA as well as the cell line used. In agreement with previous reports, the distribution of indels at a given target site was reproducible [30]. CRISPR/Cas9 editing at a DNA level caused a significant decrease of the LT protein, especially in WAGA cells, in line with the higher transfection and cleavage efficiencies. CRISPR/Cas9 editing could not be reliably evaluated at the RNA level. LT-sgRNA might also affect sT expression to some extent, due to overlapping 3′-coterminal transcripts.

As previously reported [31], the downregulation of LT protein resulted in the impaired proliferation of MCPyV^+^ cells. The antiproliferative effects were not observable in HEK293T cells, which are MCPyV^−^, suggesting that the TAs-targeting sgRNAs did not exert off-target activity affecting cell proliferation.

Consistent with previous findings [21], the slow-growing MCPyV^+^ cells exhibited a prominent peak in the G1 phase. When the expression of TAs was impaired, WAGA cells presented a significant reduction in cell cycle progression. Though the same was suspected to occur in MS-1, no significant changes were observed among the different conditions. Importantly, neither cells expressing the non-targeting ctr-sgRNA nor HEK293T cells expressing the TAs-targeting sgRNAs showed this reduction in cell cycle progression, suggesting a cell cycle arrest upon CRISPR/Cas9 targeting of TAs.

As evidenced by the cell death profiles in WAGA cells, electroporation, and presence of exogenous DNA caused certain damage in MCPyV^+^ cells, including cells expressing the ctr-sgRNA. Nevertheless, they progressively recovered to the levels of the cell control, contrary to those cells expressing the TAs-targeting sgRNAs. This observation could also explain the delay in cell proliferation. HEK293T cells were transfected with a lipid-based method, which requires a lower amount of DNA and is less cytotoxic.

**Figure 7:**
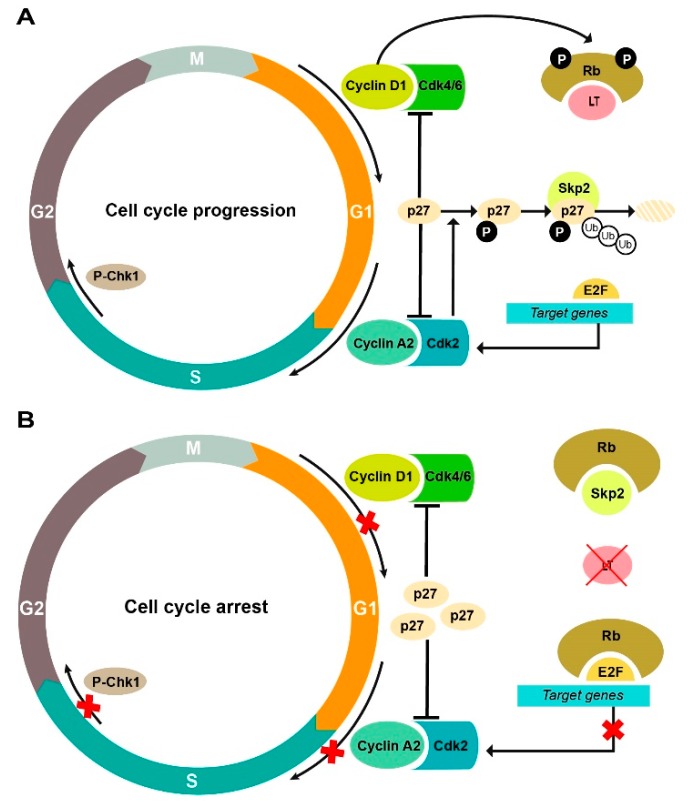
Schema of cell cycle regulation by MCPyV LT. (**A**) When MCPyV LT is present, high levels of cyclins and cyclin-dependent kinases promote cell cycle progression and Rb-phosphorylation. Cdk2 also phosphorylates the negative regulator p27, which is recognized by Skp2 for subsequent degradation; (**B**) Upon CRISPR/Cas9 editing of MCPyV LT, the Rb protein can repress the expression of the E2F target genes, contributing to cell cycle arrest. It can also interact with Skp2, promoting the accumulation of p27 to inhibit cell cycle progression.

During normal G1/S progression, cyclin-dependent protein kinases (Cdks)-mediated phosphorylation of Rb inhibits its binding to the transcription factor E2F [32]. Nevertheless, proteins containing the conserved LXCXE motif can bind Rb and reduce Rb–E2F complex formation, promoting the expression of E2F target genes [33] (Figure 6B). LT from all polyomaviruses contain this motif [6], yet Rb-binding is not unique to polyomaviruses, i.e., HPV E7 [34]. Thus, the effects of CRISPR/Cas9 editing of MCPyV TAs could be further explained by alterations in the Rb-E2F pathway (Figure 7). We found a marked decrease in products of E2F target genes that promote S phase entry: cyclin A2, Cdk2, and survivin. P-Chk1 (phosphorylated checkpoint kinase 1), another E2F-responsive element that accumulates in S phase to ensure successful DNA replication, was also significantly decreased [35]. Rb phosphorylation is opposed by several Cdk-inhibitors (CKIs) such as p27 [36]. In normal cells, Rb inhibits the ubiquitin-mediated degradation of p27, which promotes cell cycle arrest [37]. In accordance with this model, TAs knockdown resulted in increased levels of p27. On the contrary, Cyclin D1 and Cdk6 were decreased. Cyclin D1 induces Rb-phosphorylation and sequestration of p27 [36]. Generally, these findings provide strong evidence that alterations in cell cycle regulators upon TAs knockdown contribute to cell cycle arrest.

Our findings were restricted by the efficacy to transiently transfect MCPyV^+^ MCC cells and edit the viral TAs. Hence, MS-1 cells experienced lower transfection efficiency and higher electroporation-induced cytotoxicity, resulting in the slight effects of TAs editing. The introduction of specific modifications with CRISPR/Cas9 might allow targeting each MCPyV TA individually to elucidate their respective functions in MCC. However, targeting only the sT could also affect the levels of LT protein, due to lack of the LSD domain.

The trend of increasing incidence of MCC is expected to persist, owing to the aging of the population with prolonged UV-light exposure and immunosuppression [1]. Moreover, a mortality rate between 33–46% makes MCC one of the most aggressive types of skin cancer [19]. In view of the majority of MCCs being caused by MCPyV, viral TAs are an attractive target for a therapeutic CRISPR/Cas9 strategy. Currently, efforts are being made for the development of a CRISPR/Cas9-based therapeutic tool against viruses causing diseases, such as HPV [38] and HIV [39], and to treat hereditary genetic disorders [40,41].

In summary, we report the use of CRISPR/Cas9 to target MCPyV TAs in MCC. Our data confirmed that MCPyV TAs have a crucial role in the maintenance of MCPyV^+^ MCC cells. In addition, we obtained insights into the effects of the TAs on cell cycle regulators, which could aid to identify targets for novel therapies for MCC, e.g., agents that avoid p27 degradation [42]. Future experiments should focus on the development of a safe and efficient delivery system, especially in cases where the lesions are not directly reachable [43,44].

## 4. Materials and Methods

### 4.1. Cell Lines

MS-1 was obtained from the European Collection of Authenticated Cell Cultures (ECACC Cat#09111802). The WAGA cell line was kindly provided by Roland Houben (University Hospital Würzburg, Germany). These cells were tested for mycoplasma contamination (MycoAlert™ detection kit, Lonza, Verviers, Belgium) and grown in RPMI 1640 + GlutaMAX^TM^-l medium supplemented with 20% FBS. MCPyV^+^ MCC cell lines grow as cell suspensions: WAGA grow as single-cell suspensions but MS-1 necessarily needs to form spheroid cell clusters to maintain cell viability. For this reason, prior to analysis MS-1 cell clusters were disrupted by incubation with trypsin-EDTA (0.25%) and gelatin. HEK293T cells were maintained in DMEM with 10% FBS. Media were supplemented with 1× MEM NEAA, 1 mM sodium pyruvate 100 mM, 1× Penicillin/Streptomycin/Glutamine 100× and 10 mM HEPES 1 M. All media and supplements were purchased in Thermo Fisher Scientific (Merelbeke, Belgium).

### 4.2. Design of sgRNAs and Plasmid Preparation

CRISPR/Cas9 editing was performed using the GeneArt™ CRISPR Nuclease Vector with OFP Reporter (Thermo Fisher Scientific, Merelbeke, Belgium). Two target-specific sgRNAs with a reduced number of off-target sites were designed with CRISPRdirect (https://crispr.dbcls.jp/) [45], using the genome sequence of MS-1 (Accession no. JX045709) as input. To explore off-target activity more exhaustively, a BLAST search of the human genome was performed. The candidates showed a difference of at least more than two nucleotides with any other human genomic sequence. The sT/LT-sgRNA targets a region (nts 259–277) in the exon shared by MCPyV TAs. The LT-sgRNA targets the exon 2 of LT (nts 960–978) and upstreams the Rb-binding domain.

Target-specific oligonucleotides (all oligonucleotides used in this study are described in Table S1) were synthesized with 3’-overhangs with compatible ends for cloning into the GeneArt™ Vector. Then, the circularized vector was transformed into One Shot TOP10 chemically competent *E. coli* cells (Thermo Fisher Scientific, Merelbeke, Belgium). Ampicillin-resistant colonies were cultured overnight for DNA extraction using the Wizard^®^ Plus SV Minipreps DNA Purification System (Promega, Leiden, Netherlands). The presence of the correct insert was confirmed by Sanger sequencing, using primers flanking the cloning site of the vector (named primers OFP vector, Table S1). Following successful cloning, 50 mL midipreps were performed using the PureLink™ HiPure Plasmid Midiprep Kit (Thermo Fisher Scientific, Merelbeke, Belgium). Plasmid concentration and purity were assessed with Nanodrop ND-1000 (Isogen Life Science, Sint-Pieters-Leeuw, Belgium).

### 4.3. Transient Cell Transfection

ScreenFect A-Plus (Incella GmbH, Eggenstein-Leopoldshafen, Germany) was used to transfect HEK293T cells following the manufacturer’s instructions. For MCPyV^+^ cells, the 24-well optimization protocol of the Neon™ Transfection System 10 µL Kit (Thermo Fisher Scientific, Merelbeke, Belgium) was applied. Briefly, 2 × 10^5^ cells/condition were electroporated with 1 µg of DNA and plated in 24-well plates. Transfection efficiency was quantified by flow cytometry (BD Accuri C6, BD Biosciences, San Jose, CA, USA). Once the optimal conditions were selected, 10 µg of DNA was used to electroporate 3–5 × 10^6^ cells for further analysis using the 100 µL kit.

### 4.4. Sorting, DNA Extraction, PCR and Sequencing 

To determine the ability of the TAs-targeting sgRNAs to induce indels, OFP^+^ cells were sorted using the BD FACSAria III Cell Sorter (BD Biosciences, San Jose, CA). The rest of the experiments described in the present study were performed with pools of transfected cells, unsorted. DNA was extracted from transfected cells using the QIAamp^®^ DNA Blood Mini Kit (Qiagen, Benelux BV, Antwerpen, Belgium). FastStart^TM^ High Fidelity PCR (Roche, Mannheim, Germany) with primers flanking the sgRNAs target sites (Table S1) was used to amplify each sgRNA target region as a single amplicon. PCR cycling conditions were 95 °C for 5 min, followed by 38 cycles of 94 °C for 1 min, 59 °C for 20 s, and 72 °C for 1 min, with a final extension step of 72 °C for 5 min, performed on an Eppendorf Mastercycler ProS (Eppendorf, Hamburg, Germany).

Amplicons were sequenced using the BigDye^TM^ Terminator v3.1 Cycle Sequencing Kit (Thermo Fisher Scientific, Merelbeke, Belgium) on a Veriti^TM^ Thermal Cycler (Applied Biosystems, Foster City, CA, USA), using the same primers and the following conditions: 96 °C for 1 min followed by 25 cycles of 96 °C for 10 s, 50 °C for 5 s, and 60 °C for 4 min. Sequencing products were separated by size on an ABI 3730 Genetic Analyzer (Applied Biosystems, Foster City, CA, USA). Amplicon sequences were compared to the amplicons obtained from DNA of cells expressing the ctr-sgRNA, using the software SeqScape v2.7 (Applied Biosystems, Foster City, CA, USA). Sequencing results of at least three independent experiments were subjected to TIDE (https://tide.nki.nl/) analysis (limited to indels of size 0–5 that passed a significant cutoff, *p* < 0.001) to determine the frequencies and distribution of Cas9-induced mutations.

### 4.5. RNA Extraction and RT-qPCR

At day 3 post-transfection, 5 × 10^5^ cells were pelleted, washed with DPBS and disrupted/homogenized using QIAzol Lysis Reagent (Qiagen, Benelux BV, Antwerpen, Belgium). The rest of the protocol was performed with RNeasy Mini Kit (Qiagen, Benelux BV, Antwerpen, Belgium), including a DNase digestion step with RNase-Free DNase Set (Qiagen, Benelux BV, Antwerpen, Belgium). The RNA concentration was determined by A_260_ reading with Nanodrop ND-1000. One-step RT-qPCR was performed using SuperScript™ III Platinum™ One-Step qRT-PCR Kit (Thermo Fisher Scientific, Merelbeke, Belgium). Two primer sets amplifying the MCPyV TAs were used, with internal TaqMan probes (Table S1). The RPLP0 (Ribosomal Protein Lateral stalk subunit P0) reference gene was used as the endogenous control. Reactions were run on an ABI 7500 Fast RT-PCR System (Applied Biosystems, Foster City, CA, USA). Cycling conditions were as follows: 50 °C for 30 min, 95 °C for 2 min, followed by 45 cycles of 95 °C for 3 s and 60 °C for 30 s. Each sample, from four independent experiments, was run in triplicate. Data were analyzed using 7500 Fast System SDS software v1.4 (Applied Biosystems, Foster City, CA, USA) and to quantify mRNA expression relative to the endogenous control the 2^−ΔΔCt^ method was applied.

### 4.6. Cell Proliferation Assay

One day after transfection, cells were counted with a Coulter counter and seeded in 96-well plates (5000 cells/well for HEK293T, 10,000 cells/well for WAGA and 15,000 cells/well for MS-1). Cell growth was monitored using the tetrazolium salt WST-1 (Roche, Mannheim, Germany). After adding the reagent, plates were incubated at 37 °C for 4 h. Then, the formazan product absorbance at 450 and 690 nm was measured with a SpectraMax Plus 384 microplate reader. For each time point, three different wells were measured to determine the mean absorbance in each of at least three individual experiments.

### 4.7. Protein Extraction and Immunoblotting

Protein extracts were obtained with RIPA Buffer (Thermo Fisher Scientific, Merelbeke, Belgium) containing cOmplete™, Mini, EDTA-free Protease Inhibitor Cocktail (Roche, Mannheim, Germany). Proteins were separated by SDS-PAGE on 10% Criterion XT Bis-Tris gels (Bio-Rad Laboratories, Hercules, CA, USA), transferred to PVDF membranes and incubated overnight at 4 °C with the correspondent primary antibody. The following day, membranes were incubated with HRP-conjugated secondary antibodies for 1 h at room temperature. Peroxidase activity was detected with SuperSignal^TM^ West Femto Maximum Sensitivity Substrate (Bio-Rad, Hercules, CA, USA). Images were captured with a ChemiDoc^TM^ MP Imaging System and analyzed with Image Lab^TM^ v6 software (Bio-Rad, Hercules, CA, USA). All antibodies used in the present study are listed in Table S2. Densitometry analysis was performed with ImageJ software. Blots showing molecular weight markers and densitometry readings are included in Figures S3 and S4.

### 4.8. Cell Cycle Analysis

BD Cycletest™ Plus DNA Kit (BD Biosciences, San Jose, CA, USA) was used to stain cellular nuclei of 2 × 10^5^ cells with propidium iodide (PI), following the manufacturer’s instructions. Cell cycle phase distribution was evaluated using BD Accuri C6 flow cytometer, acquiring 2 × 10^4^ events for each sample. Data were analyzed with FlowJo v10 (Tree Star, Williamson Way, Ashland, OR, USA).

### 4.9. Apoptosis Assay

Apoptosis assays were performed at days 1, 2, 3, 5, and 8 post-transfection. Briefly, 3–5 × 10^6^ cells were pelleted and labeled with eBioscience™ Fixable Viability Dye eFluor™ 520 (Thermo Fisher Scientific, Merelbeke, Belgium). Cells heated up at 65 °C for 5 min served as positive controls. Apoptotic cells were detected with eBioscience™ Annexin V-APC (allophycocyanine) Apoptosis Detection Kit (Thermo Fisher Scientific, Merelbeke, Belgium) using BD Accuri C6. For each sample, 2 × 10^4^ events were recorded and data were analyzed with FlowJo v10.

### 4.10. Statistics

Statistical analyses (paired *t*-test) were performed with GraphPad Prism 7 (GraphPad Software Inc., La Jolla, CA, USA). Significance was defined with the following *p*-values: * *p* < 0.05, ** *p* < 0.01, *** *p* < 0.001, and **** *p* < 0.0001.

## 5. Conclusions

The results of the present study support the development of the CRISPR/Cas9 system as a therapeutic option for the treatment of patients with virus-positive MCC that do not respond to conventional treatments. Our data confirmed previous findings regarding the importance of the viral TAs to support the growth of MCC cell lines. In addition, we gained an understanding of the involvement of MCPyV LT in the mechanisms of cell cycle regulation. In summary, our results should prompt future research towards the establishment of CRISPR/Cas9 for the treatment of virus-positive MCC.

## Supplementary Materials

The following are available online at https://www.mdpi.com/2072-6694/11/9/1260/s1, Figure S1: RT-qPCR analysis of MCPyV TAs in WAGA cells after CRISPR/Cas9 targeting, Figure S2: Effect of CRISPR/Cas9 editing of MCPyV TAs on caspase 3 activation, Table S1: Name and sequence of oligonucleotides (sgRNAs, primers and probes) used in the present study, Table S2: Antibodies used in the present study, Figure S3: Blots of Cas9 and LT protein expression with molecular weight markers denoted in red, Figure S4: Blots of cell cycle regulatory proteins that were deregulated upon CRISPR/Cas9 editing of MCPyV TAs in WAGA cells.
