# Supplementary material for: CRISPR/Cas9 Editing of the Polyomavirus Tumor Antigens Inhibits Merkel Cell Carcinoma Growth In Vitro
Source: Cancers (Basel). 2019 Aug 28;11(9):1260. doi: 10.3390/cancers11091260 (PMC6770690; doi:10.3390/cancers11091260)
Supplement: Supplementary file 1 [file cancers-11-01260-s001.pdf]

Supplementary Materials:

CRISPR/Cas9 Editing of the Polyomavirus Tumor Antigens Inhibits Merkel Cell Carcinoma Growth In Vitro

Arturo Temblador, Dimitrios Topalis, Graciela Andrei, and Robert Snoeck

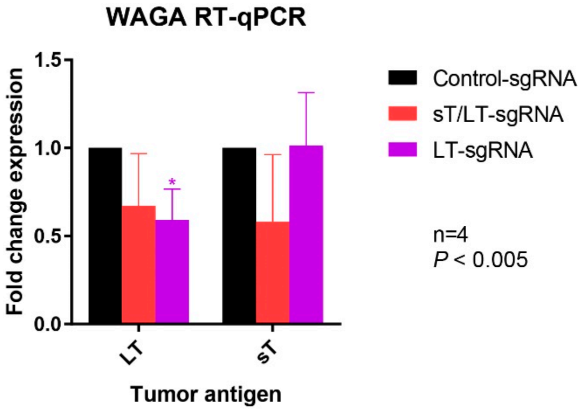

Figure S1. RT-qPCR analysis of MCPyV TAs in WAGA cells after CRISPR/Cas9 targeting. Ct values were standardized using the internal control RPLP0. Data are represented as mean values ± SD of four independent experiments. Statistical significance is indicated ($p < 0.05$).

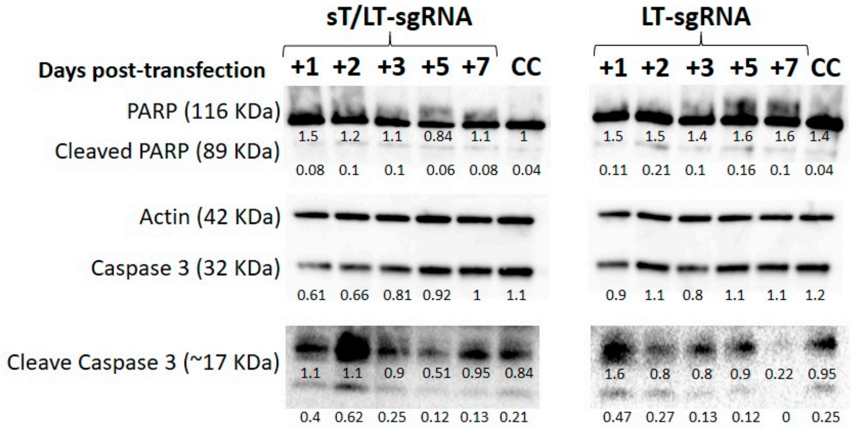

Figure S2. Effect of CRISPR/Cas9 editing of MCPyV TAs on caspase 3 activation. Total protein extracts of pools of transfected WAGA cells were used for western blot analysis of Caspase 3 and PARP. The numbers below the bands represent densitometry analysis of intensity normalized to actin.

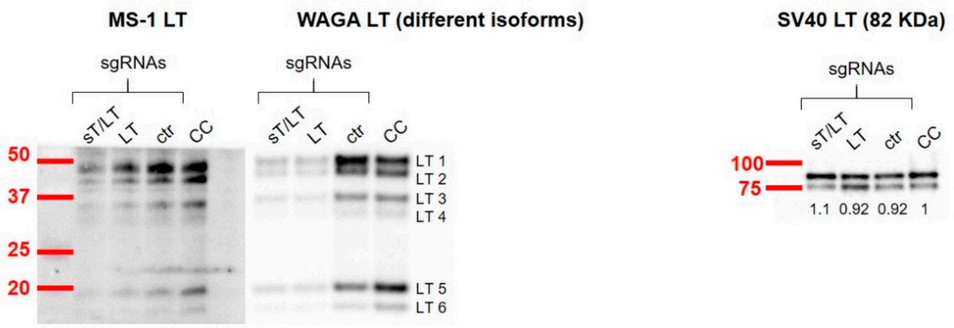

MS-1	sT/LT-sgRNA	LT-sgRNA	ctr-sgRNA	CC
LT 1	0.37	0.72	1.13	1.1
LT 2	0.2	0.38	0.71	0.85
LT 3	0.12	0.18	0.27	0.36
LT 4	0.07	0.09	0.14	0.16
LT 5	0.11	0.13	0.24	0.38
LT 6	0.11	0.04	0.06	0.12

WAGA	sT/LT-sgRNA	LT-sgRNA	ctr-sgRNA	CC
LT 1	0.31	0.27	1.38	1.11
LT 2	0.21	0.18	1.1	0.96
LT 3	0.1	0.1	0.41	0.58
LT 4	0.03	0.03	0.12	0.2
LT 5	0.11	0.16	0.72	0.94
LT 6	0.03	0.03	0.16	0.26

Figure S3. Blots of Cas9 and LT protein expression with molecular weight markers denoted in red. The numbers below the bands and in the tables represent the densitometry data (as a mean of three independent experiments) normalized to vinculin.

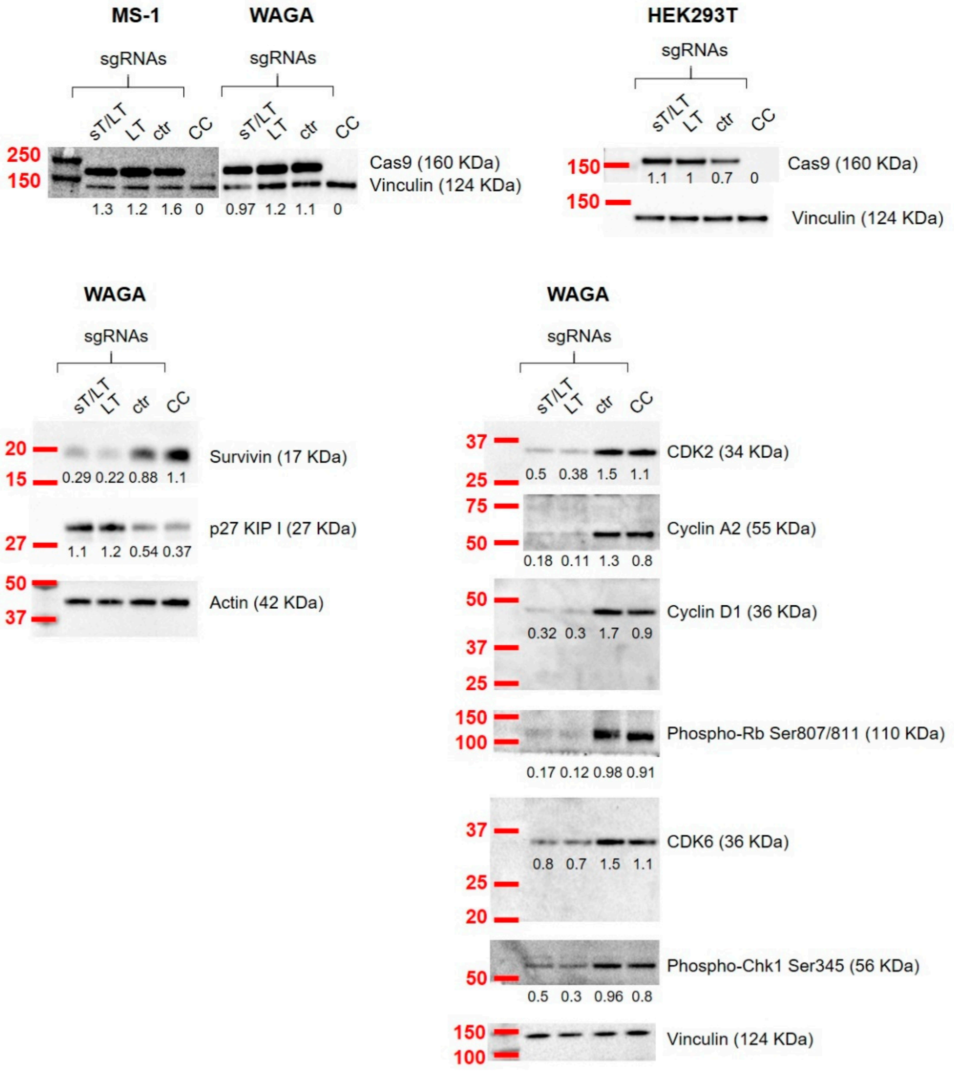

Figure S4. Blots of cell cycle regulatory proteins that were deregulated upon CRISPR/Cas9 editing of MCPyV TAs in WAGA cells. The molecular weight markers are indicated in red. The numbers below the bands represent the densitometry data (as a mean of three independent experiments) normalized to actin (left) or vinculin (right).

Table S1. Name and sequence of oligonucleotides (sgRNAs, primers and probes) used in the present study**.** All oligonucleotides were synthesized by Eurofins Genomics (Ebersberg, Germany).

Oligonucleotide name	Sequence (5′→ 3′)
sT/LT-sgRNA	GGATGTTGCCATAACAATT
LT-sgRNA	ATCTTGAGTTGGTCCCGTG
Ctr-sgRNA	TCCGTCTGCTTCATGAGCGG
Primer OFP vector forward	GAGGGCCTATTTCCCATGAT
Primer OFP vector reverse	ACCATGATTACGCCAAGCTC
Primer flanking sT/LT-sgRNA target region, forward	TAGTGAGGTGGCTCATTTGC
Primer flanking sT/LT-sgRNA target region, reverse	ATTTGCTCCAAAGGGTGTTC
Primer flanking LT-sgRNA target region, forward	GGAGCAAATTCCAGCAAAAT
Primer flanking sT/LT-sgRNA target region reverse	ATATCGGGTCCTCTGGACTG
Primer MCPyV sT, qPCR forward	GTTGTCTCGCCAGCATTGTA
Primer MCPyV LT, qPCR reverse	CCCAAGTAGGAGGAAATCCA
Primer MCPyV sT, qPCR forward	CTCTGGGTATGGGTCCTTCTC
Primer MCPyV LT, qPCR reverse	AGAGGATGAGGTGGGTTCCT
RPLP0 forward	GGCGACCTGGAAGTCCAACT
RPLP0 reverse	CCATCAGCACCACAGCCTTC
Probe sT	AAAAACTGTCTGACGTGGGGAGAGT
Probe LT	CAGTCCAGAGGACCCGATATACCTC
Probe RPLP0	ATCTGCTGCATCTGCTTGGAGCCCA

Table S2. Antibodies used in the present study.

Antibody	Source	Identifier
Mouse monoclonal anti-MCPyV LT (CM2B4)	Santa Cruz Biotechnology (Dallas, TX, USA)	Cat#sc-136172
Mouse monoclonal anti-Survivin (D-8)	Santa Cruz Biotechnology	Cat#sc-17779
Mouse monoclonal anti-V5 tag (SV5-Pk1)	Abcam (Cambridge, UK)	Cat#ab27671
Rabbit monoclonal anti-Vinculin (EPR8185)	Abcam	Cat#ab129002
Mouse monoclonal anti-SV40 T-antigen (PAb416)	Abcam	Cat#ab16879
Rabbit monoclonal anti-Actin (EPR16769)	Abcam	Cat#ab179467
Rabbit monoclonal anti-p27 KIP 1 (Y236)	Abcam	Cat#ab32034
Rabbit monoclonal anti-CDK2 (E304)	Abcam	Cat#ab32147
Apoptosis WB Cocktail	Abcam	Cat#ab136812
Mouse monoclonal anti-CDK6 (DCS83)	Cell signaling Technology (Danvers, MA, USA)	Cat#3136
Mouse monoclonal anti-Cyclin A2 (BF683)	Cell signaling Technology	Cat#4656
Rabbit monoclonal anti-Cyclin D1 (92G2)	Cell signaling Technology	Cat#2978
Rabbit monoclonal anti-Phospho-Rb (Ser807/811) (D20B12)	Cell signaling Technology	Cat#8516
Rabbit monoclonal anti-Phospho-Chk1 (Ser345) (133D3)	Cell signaling Technology	Cat#2348

Swine polyclonal anti-Rabbit Immunoglobulins	Agilent	Cat#P0399
Goat polyclonal anti-Mouse Immunoglobulins	Agilent	Cat#P0447
